# Supplementary material for: BACE1 regulates sleep–wake cycle through both enzymatic and non–enzymatic actions
Source: EMBO Rep. 2025 Nov 20;27(1):50–68. doi: 10.1038/s44319-025-00604-4 (PMC12796456; doi:10.1038/s44319-025-00604-4)
Supplement: Supplementary file 1 — Appendix [file 44319_2025_604_MOESM1_ESM.pdf]

Appendix

Table of Contents

Appendix Figure S1...................................... 2

Appendix Figure S2...................................... 2

Appendix Figure S3...................................... 3

Appendix Figure S4...................................... 5

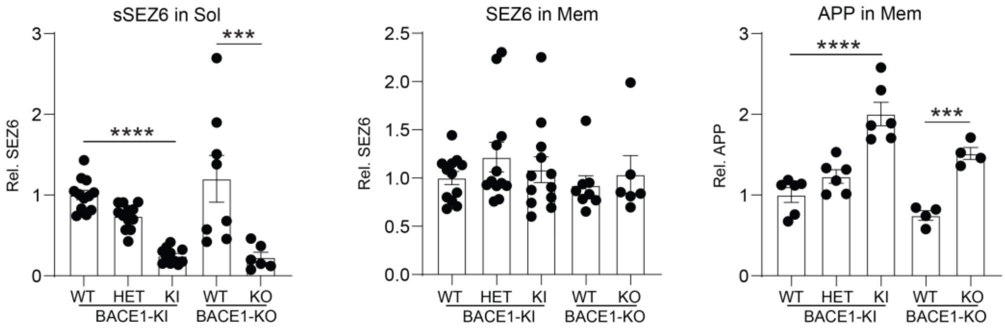

Appendix Figure S1. Quantification of SEZ6 and APP in soluble and membrane fraction from BACE1-KI and BACE1-KO mice. Data are shown with mean and SEM. n = 11-12 (SEZ6, BACE1-KI); 6 (APP, BACE1-KI); 6-8 (SEZ6, BACE1-KO); 4 (APP, BACE1-KO). One-way ANOVA with Tukey's multiple comparison test (BACE1-KI); unpaired Student's t-tests were performed for the individual comparisons (BACE1-KO) (***$p < 0.001$, ****$p < 0.0001$).

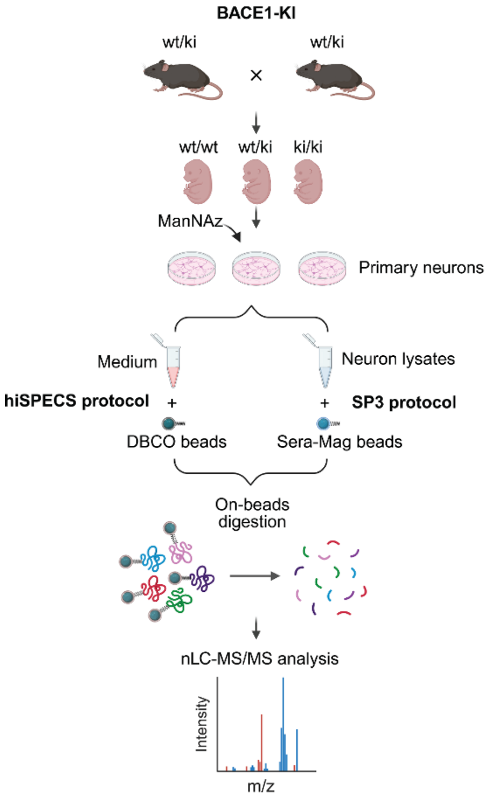

Appendix Figure S2. Proteomics analysis of secretome and lysate of primary neurons in BACE1-KI mice. Illustration of the secretome (hiSPECS) and lysate (SP3) proteomic analysis of primary neurons from BACE1-KI mice.

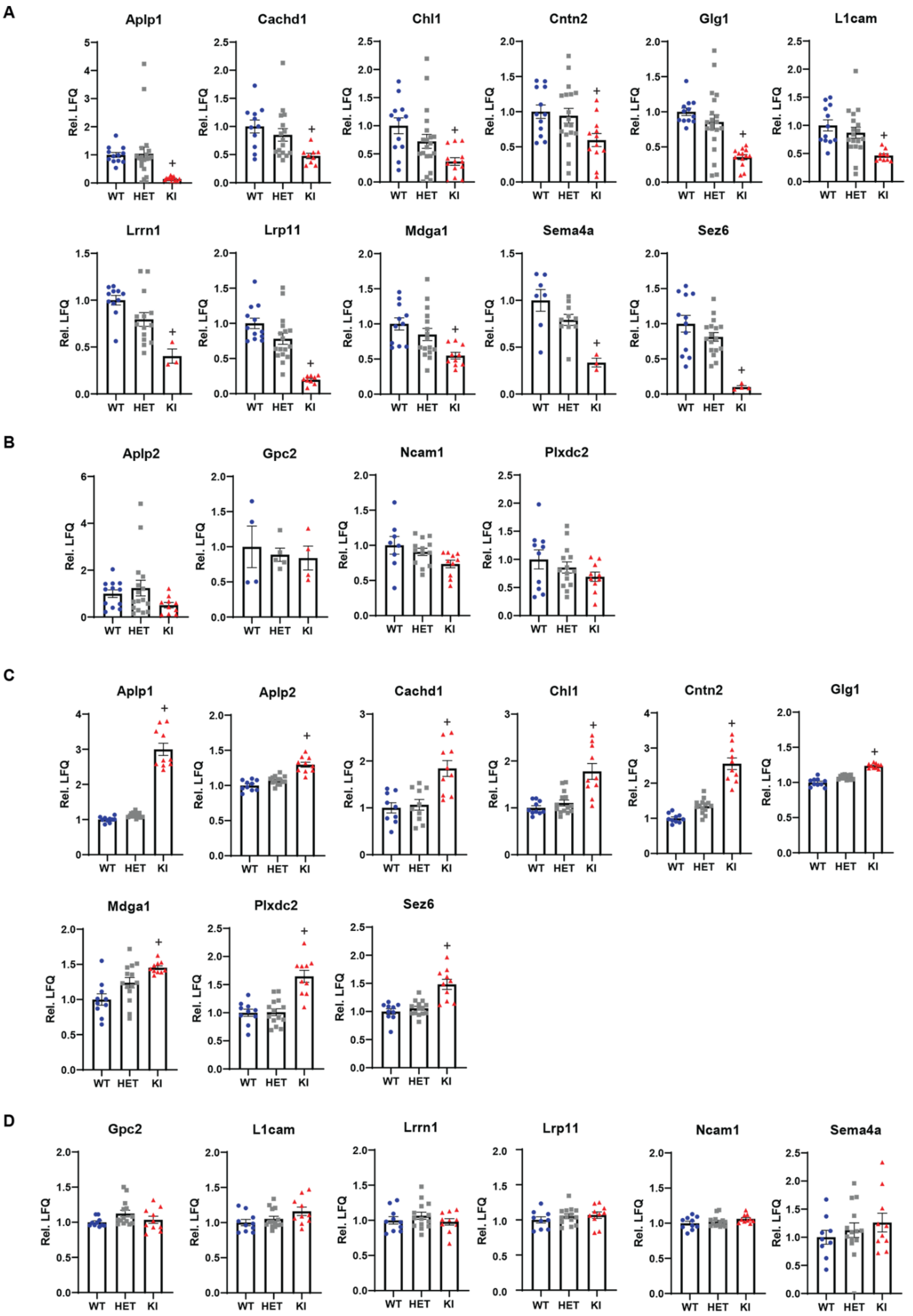

Appendix Figure S3. Scatter plots showing the relative mass spectrometry (MS) quantification normalized on the WT samples of the BACE1 substrates in neuronal

secretome and lysates of BACE1-KI mice. A,B Significant **A** and non-significant **B** alteration of relative MS quantification of BACE1 substrates in neuronal secretome analysis with the hiSPECS protocol. n = 3-20. **C,D** Significant **C** and non-significant **D** alteration of relative MS quantification of BACE1 substrates in neuronal lysate analysis with the SP3 protocol. n = 9-13. All conditions were compared against WT control, and plus signs (+) indicate significance after permutation-based FDR correction as determined in the volcano plots in Fig. 1. Data are presented as mean ± SEM, n = 6-8.

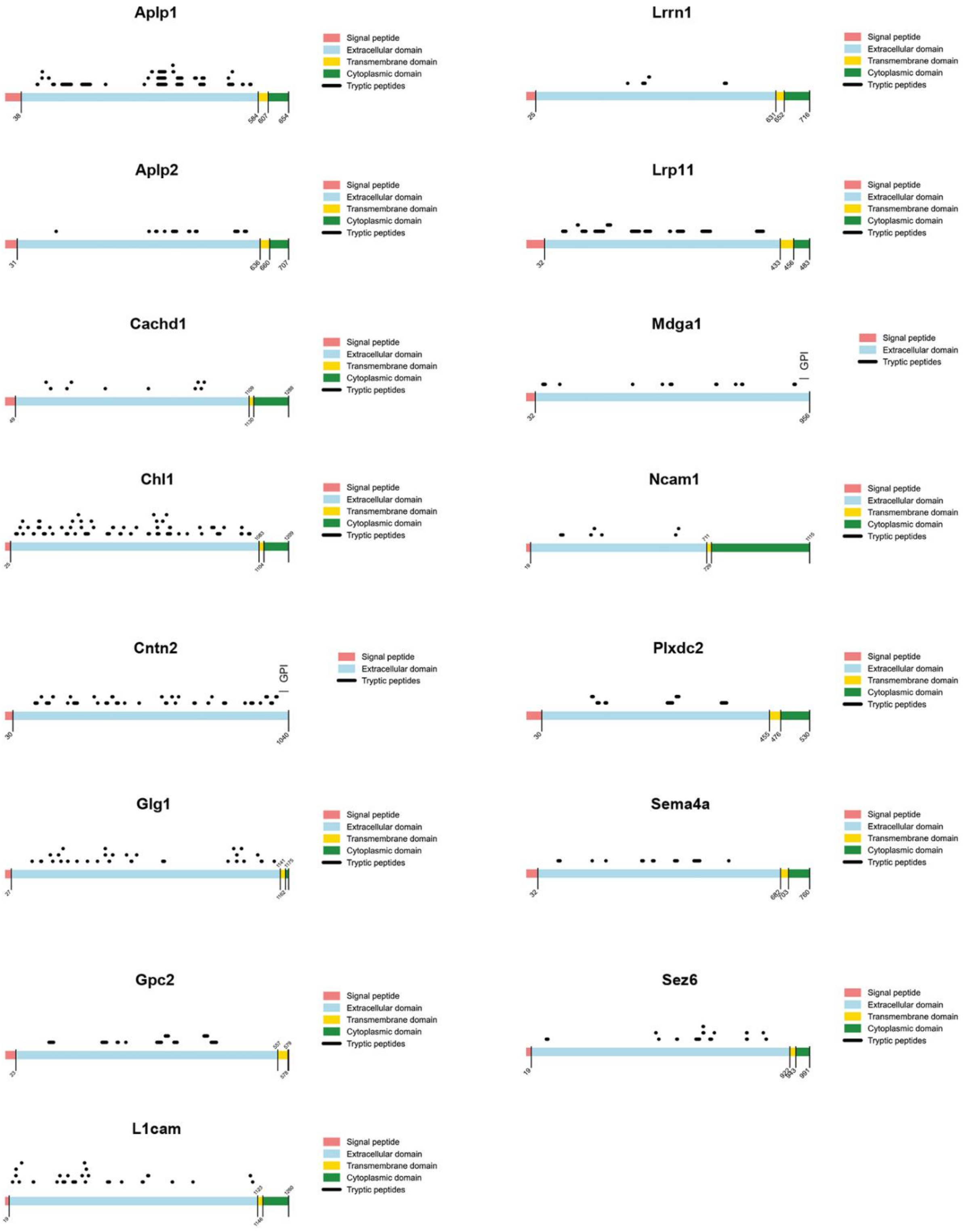

Appendix Figure S4. Peptide mapping onto the topology of BACE1 substrates in neuronal secretome analysis shows that the identified peptides are solely derived from the protein ectodomains. (pink: signal peptide, light blue: ectodomains, yellow: transmembrane domains, green: cytoplasmic domains, black: identified peptides). CNTN2 and MDGA1 have a GPI anchor as indicated.
